# Recent Developments in Sweat Analysis and Its Applications

**DOI:** 10.1155/2015/164974

**Published:** 2015-03-09

**Authors:** Saima Jadoon, Sabiha Karim, Muhammad Rouf Akram, Abida Kalsoom Khan, Muhammad Abid Zia, Abdul Rauf Siddiqi, Ghulam Murtaza

**Affiliations:** ^1^Department of Natural Resources Engineering and Management, University of Kurdistan, Hewler 44003, Iraq; ^2^University College of Pharmacy, University of the Punjab, Lahore 54000, Pakistan; ^3^Department of Pharmacy, University of Sargodha, Sargodha 40100, Pakistan; ^4^Department of Chemistry, COMSATS Institute of Information Technology, Abbottabad 22060, Pakistan; ^5^Department of Chemistry, University of Education, Attock Campus, Attock 43600, Pakistan; ^6^Department of Biosciences, COMSATS Institute of Information Technology, Islamabad 45320, Pakistan; ^7^Department of Pharmacy, COMSATS Institute of Information Technology, Abbottabad 22060, Pakistan

## Abstract

Currently, the clinical use of sweat as biofluid is limited. The collection of sweat and its analysis for determining ethanol, drugs, ions, and metals have been encompassed in this review article to assess the merits of sweat compared to other biofluids, for example, blood or urine. Moreover, sweat comprises various biomarkers of different diseases including cystic fibrosis and diabetes. Additionally, the normalization of sampled volume of sweat is also necessary for getting efficient and useful results.

## 1. Introduction

**Figure 1:**
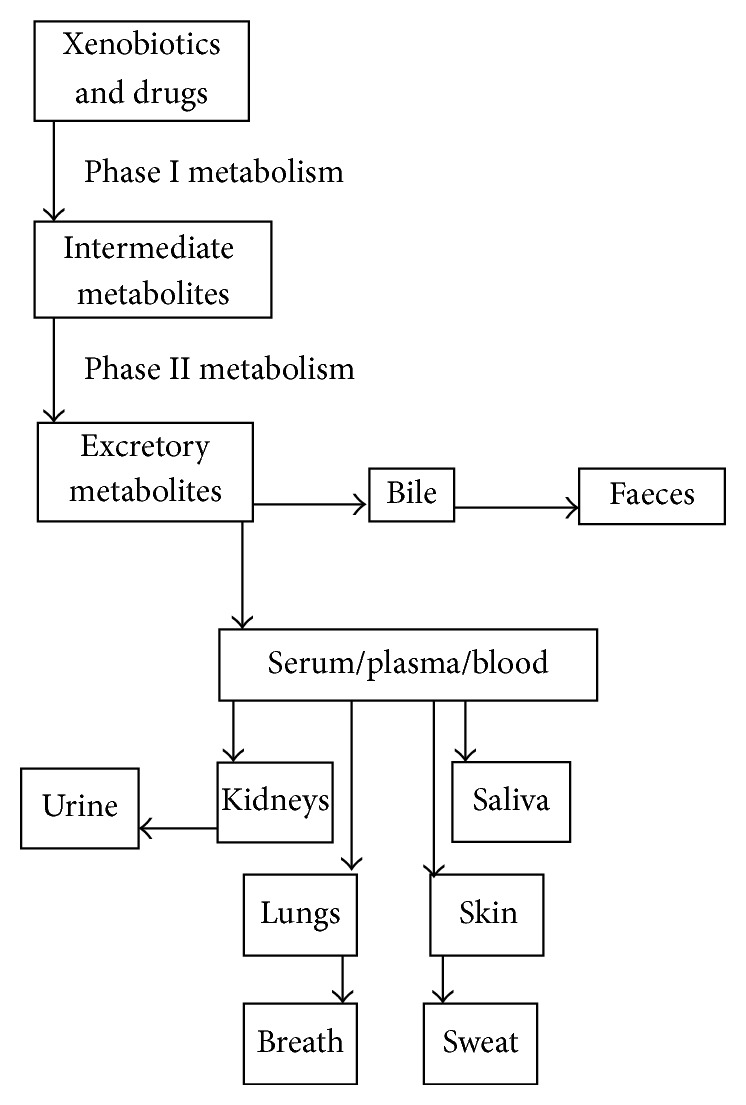
Routes of excretion of various products after liver metabolism.

Similar to the sebaceous glands or hair follicles, sweat glands are epidermal appendages that are normally distributed over the whole body, excluding the nipples, lips, and external genital organs. Sweat glands are involved in perspiration and act as excretory organ like kidney and lungs for drugs and their metabolites, as shown in Figure 1. On the basis of morphology or secretory mechanism, there are two types of sweat glands, that is, eccrine and apocrine glands. Sweat glands allow the secretion of sweat through or without pinching off of outer cell parts [1]. Sweat is normally a transparent biofluid with low tonicity and slightly acidic nature with mean pH 6.3, that is, more acidic than blood [2]. Thus, basic drugs preferably accumulate in sweat than blood, based on pH partition theory [3]. The transport of water insoluble drugs between blood and other biofluids depends on the pH of the other biofluids and the drug's pKa which are helpful in theoretical computation of the biofluid-to-plasma concentration ratio of drug using Henderson-Hasselbalch equation [3, 4]. The concentration gradient between plasma and sweat provides driving force for passive diffusion of the free fraction of drug from plasma to sweat through lipid bilayer [3–5]. The main content of sweat is water (~99%) [2]. Besides, small amounts of the following substances are also present: nitrogenous compounds such as amino acids and urea [6]; metal and nonmetal ions such as potassium, sodium, and chloride ions [3]; metabolites including lactate and pyruvate; and xenobiotics such as drug molecules [2]. In disease state, sweat may contain different ingredients as biomarkers of the particular disease [2, 3, 6]. Blood, urine, saliva, and sweat are the biofluids, which are used for clinical analyses such as pharmacokinetics study. Due to the presence of very nominal impurities, the sample preparation of sweat is very easy as compared with other biofluids. In addition, sweat samples are less prone to adulterations; thus such samples can be stored for long periods [7]. Unlike other biofluids, sweat possesses excellent features including its noninvasive sampling. Owing to this characteristic, sweat analysis is considered as a rapid and easy process in comparison to other biofluids, especially blood. Blood sampling is an invasive procedure; that is, it needs surgery. Patients, who require frequent analysis, are therefore at higher risk of infection. In addition, blood samples are further processed for plasma protein removal. In addition, the preparation of sweat sample is easier than that of urine. This merit of sweat as biofluid is particularly important in doping control where results are urgently needed [8, 9]. Beside these advantages, clinical use of sweat samples is presently limited due to high costs [10], time-consuming sampling, infectivity hazards, and the need of volume normalization [11]. In addition, the rigorous attention should be paid to the analysis of metabolic products present in sweat [12].

Due to increased interest in the clinical use of sweat, various approaches for sweat sampling and analysis have been documented. Thus, the objective for preparing this review article involved the summarization of various apparatus and techniques used for sampling of sweat and its analysis. Furthermore, the applications of sweat in clinical settings have also been discussed.

## 2. Sweat as Biofluid

### 2.1. Induction of Perspiration

Apart from sampling and analysis, the induction of perspiration is a distinct phenomenon unlike other biofluids, which are rather directly collected. The physiological factors which enhance perspiration for getting certain volume of sweat include exercise and stress, while reduced perspiration is observed in cold [1]. For receiving sweat volume adequate for subsequent analysis, there are many factors which induce perspiration for sampling objective; these factors include environmental factors (such as temperature and relative humidity), body regulatory systems (hormonal and sympathetic nervous system), diet, and certain sweat inducing chemical compound such as pilocarpine. Together with a low intensity electrical current (~3.0 mA) applied for approximately five minutes, pilocarpine is applied on small area of leg or arm for induction of sweating [37].

### 2.2. Sampling of Sweat

An ideal sampler is the one that is user-friendly and harmless to skin and quickly collects the sweat in sufficient volumes. Various versions of sweat samplers have been designed and used for sweat sampling. The simplest sampling technique involved the use of an occlusive patch consisting of 2-3 layers of filter paper or gauze. However, this approach was time-consuming and difficult to adopt owing to large size of patch. Moreover, these patches were nonadjustable to skin [38]. The variation in sample pH and irritation to skin were other drawbacks of this method [18]. To avoid skin irritation, nonocclusive device was prepared using Whatman filter paper patch fixed on surgical dressing film lined with an adhesive layer for adjusting to arm skin. This patch was safer to the underneath skin because of selective transfer of water, oxygen, and carbon dioxide through this semipermeable film, which hindered penetration of nonvolatile substances [38]. Since these patches allowed evaporation of water from concentrated sweat leaving sweat components such as chlorides, the entire volume of secreted sweat was not known. Resultantly, from the collected samples, the results for cystic fibrosis (CF) test were not authentic. Later on, the problem of water evaporation was solved by using air- and water-tight, sweat collection bag connected with adhesive rubber. This advanced device was capable of retaining the whole volume of secreted sweat [39]. Moreover, the design of this device was further modified by connecting it with glass rollers, pipettes, and holders to make it suitable for collection of sweat secreted in limited volumes [40].

For efficient analysis, the sweat samplers are usually associated with the analytical instruments [41]. Macroduct and Megaduct are the most frequently used commercial samplers, available in different volumes (15 *μ*L–0.5 mL). Macroduct and Megaduct are used for collection of smaller (15 *μ*L) and larger (0.5 mL) volumes of sweat, respectively. Both tools contain a concave disk and a spiral plastic tube for sweat collection [42]. Another modern sweat-sampling device is microstrip impregnated with a dye pH indicator that is a smart-phone application for* in situ* colorimetric testing [43].

### 2.3. Sample Preparation for Analysis

Generally, sweat is directly analyzed; however, sweat samples can further be processed if lipid or protein moieties are detected in sweat. After sample collection, various procedures, including liquid-liquid extraction and solid-phase extraction, for the preparation of samples are used. Liquid-liquid extraction involves the use of analyte-specific solvents (e.g., aqueous phosphate buffer and organic solvents, e.g., methanol and acetonitrile) [37]. Solid-phase extraction involves the use of cartridges that internally consists of copolymers [8]. Sometimes the drugs, xenobiotics, and electrolytes are adequately discriminatory at the detector, and then their derivatization is also carried out during sample preparation for appropriate detection or separation [44].

### 2.4. Analysis of Sweat

The quality of sweat analysis depends on the efficiency of sample collection and the accuracy and sensitivity of analytical methods [2–5, 9, 42]. Currently, different analytical approaches have been used for the analysis of unmetabolized drugs in sweat; however further focus of researchers is needed for studying drug metabolites present in sweat normalized with its volume. Most of the sweat analyzers work on the principle of potentiometry, colorimetery, conductivity, or osmolarity [43].

On coupling with mass spectrometers (MS), capillary electrophoresis and chromatographic approaches such as liquid-chromatography (LC) and gas-chromatography (GC) are valuable for high-resolution separation of drugs or complex metabolites in sweat. For drug analysis in sweat, the most frequently used equipment is GC-MS coupled with electron impact ionization [7–9, 12, 37, 44–46]. Moreover, LC-MS/MS coupled with electrospray ionization has also been successfully used [37, 47] to determine drug concentration in sweat. In this context, immunoassay approaches including radioimmune analysis and ELISA are also being employed [8, 12, 37, 45].  Table 1 describes some approaches for separation and detection-determination of drugs of abuse in sweat [8, 10, 13–19].

To analyze sweat, the analytical instrument is selected in accordance with the nature of target analyte(s) such as sodium or chloride ions [48]. For single moiety analysis, some of the most frequently used dedicated analyzers are the potentiometric Orion skin ion selective electrode (ISE) for chloride (Orion Research, Cambridge, MA), or the colorimetric Scandipharm CF Indicator System chloride patch (Scandipharm, Birmingham, AL), the Wescor Sweat-Chek conductivity analyzer (Wescor, Logan, UT), and sweat osmolarity analyzer (Nikon Research, Cambridge, ML) [49].

Due to variable volumes of sweat samples, the normalization of sweat volume is also necessary for getting authentic results [6]. For normalizing the sweat volume, Appenzeller and coauthors introduced the concept of internal standard by using and determining the level of sodium and potassium using capillary zone electrophoresis linked with diode array detector set at 214 nm [39]. However, a study has suggested that sodium ion concentration is more suitable for using in the normalization of sampled volume of sweat than potassium ion concentration [50]. However, the concept of normalization of sampled volume of sweat has not yet been applied for the diagnosis of CF.

## 3. Applications of Sweat Analysis

### 3.1. Diagnosis of Diseases

Since last three decades, much attention has been paid towards application of sweat in disease diagnosis. The best example of a disease diagnosed through sweat analysis is cystic fibrosis (CF). This disease originates from genetic transformations in CFTR proteins (cystic fibrosis transmembrane conductance regulating proteins) in the sweat gland. The CFTR proteins are, normally, responsible for the transport of sodium and chloride (transport_Na-Cl_) in epithelial secreting cells. The genetic modification of CFTR causes the change in transport_Na-Cl_ resulting in formation of sticky mucus in various organs such as lung, intestine, and other organs. This condition leads to serious repeated infections of the pancreas affected organs. Moreover, male infertility and dehydration are also observed in CF patients. The sweat analysis for sodium/chloride ratio (sodium and chloride contents) can, therefore, be useful in diagnosing CF. In particular, chloride level is disturbed in CF due to mutation in CFTR, and thus sweat chloride can be referred to as the biomarker for CF diagnosis [51, 52]. On the basis of this biomarker, there are two types of CF, that is, typical and atypical CF. With a minimum of one phenotype appearance, chloride contents ≥ 60 mmol per liter of sweat indicate typical (real positive) CF while atypical CF is manifested with chloride concentration in borderline range of 30–60 mmol per liter of sweat. The normal (and borderline) concentrations of chloride in sweat are <30 mmol/L (30–59 mmol/L) and <40 mmol/L (40–59 mmol/L) in infants and elders [51]. Moreover, sweat potassium as another biomarker is the focus of current research for early diagnosis of CF that assists in treatment efforts for the diseased person; however clinical application of this new biomarker is still under investigation [53].

On the basis of sweat analysis, diabetic biomarkers have also been reported such as the average change in sweat rates [54], composition of human sweat [40], and correlation between sweat glucose and blood glucose. The later approach produces promising results provided sweat gains no glucose from environment [55]. This diagnostic technique involves the analysis of foot sweat using a simple, reproducible indicator test [56] on the basis of color change of a patch from blue (due to the presence of anhydrous cobalt-II-chloride) to pink at 10 min on adding 6 water molecules.

Jurado Gámez et al. have proposed sweat-based diagnostic assay for lung cancer by discriminating between metabolomics of healthy and diseased subjects. In this promising approach, sweat is diluted with 0.1% formic acid followed by the injection of sample into LC-TOF/MS which necessitates only 10 *μ*L of sweat [57].

Genomics and proteomics have played a key role for searching sweat biomarkers such as dermcidin (DCD). Sweat contains DCD, a peptide containing 47-amino acids, which possesses antimicrobial activity against different pathogens in high salt concentrations and over an extensive pH range resembling to the human sweat. For this reason, sweat is considered to be crucial for human skin microflora [58]. Moreover, DCD and the receptors for DCD are present and overexpressed on the cell surface of invasive breast carcinomas and their lymph node metastases and neurons of the brain. These findings reveal that DCD is involved in tumorigenesis by promoting cell growth and survival in breast carcinomas [59]. Another prognostic biomarker is prolactin inducible protein (PIP) which is expressed in many exocrine tissues including sweat glands and is overexpressed in metastatic breast and prostate cancer [60]. In addition, prognostic biomarkers have also been investigated in a study performed on eccrine sweat in healthy and schizophrenic patients. The eccrine sweat contains plenty of various proteins and peptides unlikely to that of serum showing that eccrine sweat may produce distinctive disease-linked biomolecules [6].

### 3.2. Assessment of Drugs and Ethanol in Sweat

Currently, sweat analysis for drug contents is accomplished through two approaches, that is, early and late testing. First methodology involves the immunochromatographic testing for qualitative detection of recently used drugs (within 24 h) involving sweat sample collected at single time point for identifying the individuals who are under the effect of drugs. Second methodology involves the patch technology for qualitative detection of previously used drugs (within 168 h) involving sweat sample collected at single time point for the follow-up of drug users under treatment to substantiate abstinence [3, 61, 62].

Together with urine, sweat is an ideal sample for doping control. The volume of sweat perspired by the whole human body in one day is 300–700 mL. This biofluid contains a small but quantifiable percent of a drug [63] excreted through transcellular and paracellular pathways in skin [11, 64]. The reported drugs excreted through sweat in a quantifiable fraction are the opiates, buprenorphine, amphetamines, gamma hydroxybutyrates, cocaine, and cannabinoids [9, 65]. In addition, ethanol contents in sweat as a function of time have also been successfully analyzed after ingesting ethanol [51].

### 3.3. Assessment of Metals, Ions, and Salts in Sweat

Xenometabolomics is a branch of science that deals with the study of essential metals and xenometals in the organism contaminated through either ingestion of food or absorption through skin by occupational exposures [66]. After getting into body, some metals are converted to their xenometabolites (cations or salts) followed by their solubilization in sweat. In addition to excretion of metals as their free metals, ions, or simple salts, excretion of some metals occurs in the form of their complexes. For example, lead complexed with high molecular weight compounds excretes through sweat [25]. The excreted sweat concentrations of some metals (e.g., cadmium and lead) or their cations, salts, or complexes are sometimes comparable to those of urine; thus sweat can be used as a biofluid alternate of urine, particularly in some kidney disease [29, 34].  Table 2 elaborates the studies of metal excretion in sweat conducted in different countries. It can therefore be stated that perspiration is a potential route for the excretion of toxic metals from the body.

### 3.4. Assessment of Volatile Organic Compounds in Sweat

Large number of different compounds has been recognized in human sweat, out of which >500 compounds are volatile in nature [67]. Because of heterogeneous distribution of various sweat glands in skin, the profiles of volatile organic compounds (VOC) are different in different body regions, which also affect the odor of an individual [68, 69]. Moreover, VOC from personal care products and sweat may also interfere with each other during sweat analysis [70, 71]. In addition, compounds which are volatile at body temperature are directly collected, while the other substances are obtained through volatilization of collected sweat.

## 4. Conclusion

Based on sweat analysis, advancements in the genomics and proteomics have enormously contributed to the field of metabolomics and the systems biology. The metabolisms of the macromolecules in sweat glands produce lower molecular weight metabolites, such as the conversion of proteins to peptides or amino acids. Since, metabolomics deals with measurements of both precursor and metabolites, sweat can be used as a biofluid, in addition to blood and urine, to explore biomarkers for various diseases. Subsequently, these discoveries help in exploring effective therapeutic moieties. Since sweat consists of various biomarkers, these biomarkers have played an excellent role in diagnosis of cancer, diabetes, schizophrenia, and cystic fibrosis. Conclusively, sweat can be used as a promising biofluid for disease diagnosis and drug analysis.

## Figures and Tables

**Table 1:** Some approaches for separation and detection-determination of drugs of abuse in sweat.

Number	Analytical approach	Examples of some analyzed drugs	Limit of quantification (ng per patch)	References
1	GC-MS (electron ionization)	Cocaine, codeine, 6-acetylcodeine, morphine, 6-acetylmorphine, and heroin	5–10	[8]
2	GC-MS (electron ionization)	Cocaine, codeine, 6-acetylcodeine, morphine, 6-acetylmorphine, and heroin	5	[13]
	GC-MS (electron ionization)	Methadone	50	[14]
3	GC-MS (electron ionization)	Cocaine, codeine, 6-acetylcodeine, morphine, and heroin	50	[15]
4	GC-MS (electron ionization)	Codeine, morphine, and 6-acetylmorphine	2.5	[16]
5	GC-MS (electron ionization)	Cocaine and heroin	Not mentioned	[17]
6	GC-MS (electron ionization)	Cocaine, codeine, morphine, and 6-acetylmorphine	2.5	[18]
7	ELISA and GC-MS (electron ionization)	Codeine, morphine, 6-acetylmorphine, and heroin	3–5	[10]
8	LC-MS-MS (electrospray ionization)	Fentanyl	0.09	[19]

**Table 2:** Studies of metal excretion in sweat.

Number	Metals	Country of study subjects	References
1	Mercury	Canada	Genuis et al., 2011 [20]
2		USA	Robinson and Skelly, 1983 [21]
3		USA	Sunderman 1978 [22]
4		USA	Lovejoy et al., 1973 [23]

5	Cadmium	Australia	Stauber and Florence, 1988 [24]
6		Australia	Stauber and Florence, 1987 [25]
7		USA	Robinson and Weiss, 1980 [26]
8		USA	Cohn and Emmett, 1978 [27]
9		Canada	Genuis et al., 2011 [20]
10		UK	Omokhodion and Howard, 1994 [28]

11	Arsenic	Canada	Genuis et al., 2011 [20]
12		Bangladesh	Yousuf et al., 2011 [29]

13	Lead	Australia	Lilley et al., 1988 [30]
14		Australia	Stauber and Florence, 1988 [24]
15		Australia	Stauber and Florence, 1987 [25]
16		Canada	Genuis et al., 2011 [20]
17		UK	Omokhodion and Crockford, 1991 [31]
18		UK	Omokhodion and Crockford, 1991 [32]
19		USA	Cohn and Emmett, 1978 [27]
20		USA	Hohnadel et al., 1973 [33]
21		Tropics	Omokhodion and Howard, 1991 [34]
22		Russia	Parpaleĭ et al., 1991 [35]
23		Germany	Haber et al., 1985 [36]
